# A Brief Smoking Cessation Intervention for Heavy Drinking Smokers: Treatment Feasibility and Acceptability

**DOI:** 10.3389/fpsyt.2018.00362

**Published:** 2018-08-10

**Authors:** Aaron C. Lim, Kelly E. Courtney, Nathasha R. Moallem, Vincent C. Allen Jr., Adam M. Leventhal, Lara A. Ray

**Affiliations:** ^1^Department of Psychology, University of California, Los Angeles, Los Angeles, CA, United States; ^2^Department of Psychology, University of California, San Diego, San Diego, CA, United States; ^3^Department of Preventive Medicine and Psychology, University of Southern California, Los Angeles, CA, United States; ^4^Department of Psychiatry and Biobehavioral Sciences, University of California, Los Angeles, Los Angeles, CA, United States

**Keywords:** heavy drinking smokers, brief behavioral counseling, smoking cessation, alcohol reduction, motivational interviewing

## Abstract

Approximately 20–25% of regular smokers report heavy drinking. Abstinent smokers are five times as likely to experience a smoking lapse during drinking episodes. Current efforts seek to improve treatments for this subgroup of heavy-drinking smokers. This study tested the feasibility and acceptability of addressing alcohol use in a brief, single session smoking cessation intervention (SMK+A) compared to smoking cessation counseling only (SMK); these interventions were grounded in a motivational interview framework and included personalized feedback, decisional balance, quit day setting, and tailored skills building (e.g., breathing techniques, coping with urges, dealing with social pressures) to maintain abstinence. Descriptive outcomes included reported helpfulness of intervention skills, readiness to change scores, and feasibility of participant recruitment and retention. We also assessed 7-day point prevalence of smoking cessation, and smoking and drinking reduction at 1-month follow-up. Participants (*N* = 22) were community-based treatment-seeking daily smokers (≥5 cigarettes/day) who were also heavy drinkers (≥14 drinks/week for men, ≥ 7 drinks/week for women; or ≥5 drinks on one episode in past week for men, ≥4 for women). Twenty five percent of interested individuals were eligible after initial phone screen, and all randomized participants were retained through follow up. All skills demonstrated high acceptability (i.e., rated between moderately and very helpful), and a significant proportion of participants in each condition reported taking action to reduce cigarette smoking and/or alcohol use at 1-month post-quit. Three participants in each condition (27.3%) attained bioverified (CO ≤ 4 parts per million and cotinine ≤ 3 ng/mL) smoking quit at follow-up. Given the modified intervention's acceptability and flexibility, larger studies may help to elucidate this intervention's effects on readiness to change, smoking cessation, and alcohol reduction.

## Introduction

Despite overall declines, cigarette smoking remains prevalent with 15.1% of adults in the United States reporting regular smoking, defined as smoking at least 100 cigarettes during their lifetime and smoking at minimum several days a week (1). One problematic comorbidity that may contribute to the consistently high percentage of adult cigarette smoking prevalence in the United States is the frequent co-use of alcohol and cigarettes. Approximately 20–25% of regular smokers report heavy drinking (2–4), and abstinent smokers are five times as likely to experience a smoking lapse during drinking episodes (5). While the literature has suggested that greater alcohol use is associated with a greater likelihood of a failed smoking cessation attempt (6, 7), the frequency of heavy drinking (defined as >3 drinks on any day or ≥7 drinks per week for women and >4 drinks on any day or ≥14 drinks per week for men, according to the U.S. National Institute of Alcohol Abuse and Alcoholism) in particular appears to be more prognosticative of poor cessation outcomes than frequency of drinking more generally across the overall continuum of drinking levels (2, 5). Laboratory studies have shown that even smokers who drink at moderate levels are less able to resist smoking a cigarette after consuming alcohol, relative to a placebo beverage (8). Therefore, efforts to address smoking cessation among heavy drinking smokers may be more successful by addressing both alcohol and smoking within the same intervention.

The guidelines for smoking cessation counseling consistently describe the combined risks of smoking and drinking (9) and suggest that drinking reduction or abstinence should be recommended in order to promote a successful quit attempt (10, 11). Nevertheless, specific strategies for helping heavy-drinking smokers address their alcohol use in the context of a smoking cessation trial have not been sufficiently evaluated and developed. Recent research has recognized the need to concomitantly address alcohol use in the context of smoking cessation, particularly among smokers who are identified as heavy drinkers. In the behavioral treatment literature, Kahler et al. (12) conducted a randomized clinical trial of transdermal nicotine patch with either a standard four-session smoking cessation treatment (ST-only) or the standard treatment incorporating brief alcohol intervention (ST-BI). The ST-only intervention included addressing situations that represented a high risk for relapse, as well as encouraging efforts to seek additional treatment and support, while ST-BI incorporated normative feedback regarding the participant's smoking and drinking levels, a discussion of alcohol as a relapse risk factor, and developing drinking goals. Results revealed that participants in the ST-BI condition reported 20% fewer drinks per week and greater smoking abstinence, than individuals in the ST-only condition, over the course of the 26-week follow-up (12); the investigators noted, however, that lasting smoking cessation outcomes may require additional treatment development. Similarly, a study of tobacco quitline callers found that a brief 2-session alcohol intervention for hazardous-drinking callers resulted in higher smoking cessation rates (13), suggesting that expanding the alcohol content of even brief counseling encounters can have substantial impacts on smoking behavior.

One potential barrier for effective and broadly implemented treatments for heavy-drinking smokers is that smoking and drinking topics are often approached differently by treatment providers. Within primary care settings, physicians dedicate significantly greater amounts of time addressing smoking relative to alcohol effects on health (14, 15). Thirty percent of individuals are screened for an alcohol or drug use problem during a primary care visit (16), whereas 80% of patients report an evaluation of smoking (17). A qualitative study also found that providers tended to provide more vague recommendations regarding drinking patterns, in contrast to smoking-related recommendations (18). Multiple barriers also prevent thorough discussions about drinking, such as the sensitivity of alcohol misuse as a discussion topic, the patient's lack of awareness of the severity of their drinking, and lack of availability of evidence-based interventions (19). On the other hand, large-scale programs that funnel patients toward appropriate levels of alcohol and illicit substance use treatment, including brief counseling (e.g., Screening, Brief Intervention, and Referral to Treatment (SBIRT); (20), do not include treatments for nicotine dependence. Importantly, heavy-drinking smokers frequently perceive that their alcohol consumption increases their smoking, and report a preference for an integrated treatment modality (21). Therefore, there is a great need for interventions that address comorbid tobacco and alcohol use across treatment contexts, from multisession treatment regimens to brief face-to-face encounters with healthcare providers.

In summary, heavy-drinking smokers represent a unique subgroup of smokers for whom the available interventions may not be optimal. Current efforts seek to improve upon the standard of care for this sizeable and hard-to-treat subgroup of smokers who drink heavily. Empirically validated brief behavioral interventions have demonstrated short-term efficacy in simultaneously reducing alcohol use and increasing smoking cessation rates among heavy-drinking smokers. No study to date, however, has consolidated these smoking cessation interventions that address alcohol use into a single session of counseling. Examining the impact of such an intervention on smoking and drinking outcomes may be important for two reasons. First, addressing substance use that frequently co-occurs increases ecological validity and relevance of smoking intervention content. Second, this single-session intervention may be informative for emerging and versatile brief smoking/alcohol treatments that can be delivered across multiple treatment settings by different types of providers (22, 23). Such treatments have been shown to be cost-effective, highly feasible, and successful in reducing substance use (24).

To that end, this pilot study tested whether addressing alcohol use during a brief, single session, smoking cessation counseling (SMK+A) would be feasible and acceptable among heavy drinking smokers as compared to smoking cessation counseling only (SMK). These interventions were grounded in a motivational interview framework and included personalized feedback, decisional balance, quit day setting, and tailored skills building, as such menu-based interventions are efficacious, acceptable, and feasible (25); further, the flexibility of the menu of skills provided a pragmatic way to incorporate a discussion of alcohol. Therefore, this study sought to test the feasibility and acceptability of the tailored behavioral intervention. Acceptability was assessed through participants' subjective ratings of the usefulness of skills selected in the intervention conditions, and Readiness to Change scores for smoking and drinking behaviors at baseline and follow-up. This study also provides descriptive information regarding smoking and drinking outcomes, including bioverified 7-day point prevalence of smoking cessation (i.e., abstinence for 7 days prior to 1-month follow-up), consistent with recommendations for smoking cessation studies (26, 27), as well as drinking and smoking reduction at 1-month follow-up. Consistent with principles of feasibility reporting for small trials (28), feasibility was assessed through participant retention through follow up, as well as consideration of the percentage of study completers to the number of screened and eligible heavy-drinking smokers.

## Materials and methods

### Participants and procedures

Participants (*N* = 22) were treatment-seeking smokers recruited through community advertisements. These advertisements described an opportunity to participate in a study of smoking cessation counseling to help people quit. Interested individuals contacted the lab to complete an initial phone screening to assess substance use history and exclusionary medical and psychiatric conditions; 247 individuals completed this initial screening. Inclusion criteria were: (1) between 21 and 55 years old, (2) treatment-seeking smokers without quit attempts in the past 3 months and who were willing to set a quit date in the 30 days following the intervention visit, (3) daily smokers smoking at least five cigarettes per day, verified by carbon monoxide (CO) and cotinine levels, (4) current heavy drinking as defined by the National Institute on Alcohol Abuse and Alcoholism (29), including at least 14 drinks per week or greater than four drinks on any day in the past week for men, and at least seven drinks per week or greater than three drinks on any day in the past week for women. Exclusion criteria included: (1) Having stopped smoking for at least 3 months over the past year, (2) current regular use of illicit drugs other than marijuana, and (3) history of psychotic disorders.

Eligible individuals were invited to the laboratory to complete informed consent procedures, bioverification [CO, cotinine, and breath alcohol concentration (BrAC)] and the counseling session. Participants also completed the following assessments at this laboratory visit: 30-day Timeline Follow-Back for cigarette smoking and alcohol consumption (TLFB) (30); Fagerstrom Test for Nicotine Dependence (FTND) (31); and Alcohol Use Disorders Identification Test (AUDIT-C) (32). All study procedures were approved by the local Institutional Review Board (IRB).

### Study design

**Figure 1:**
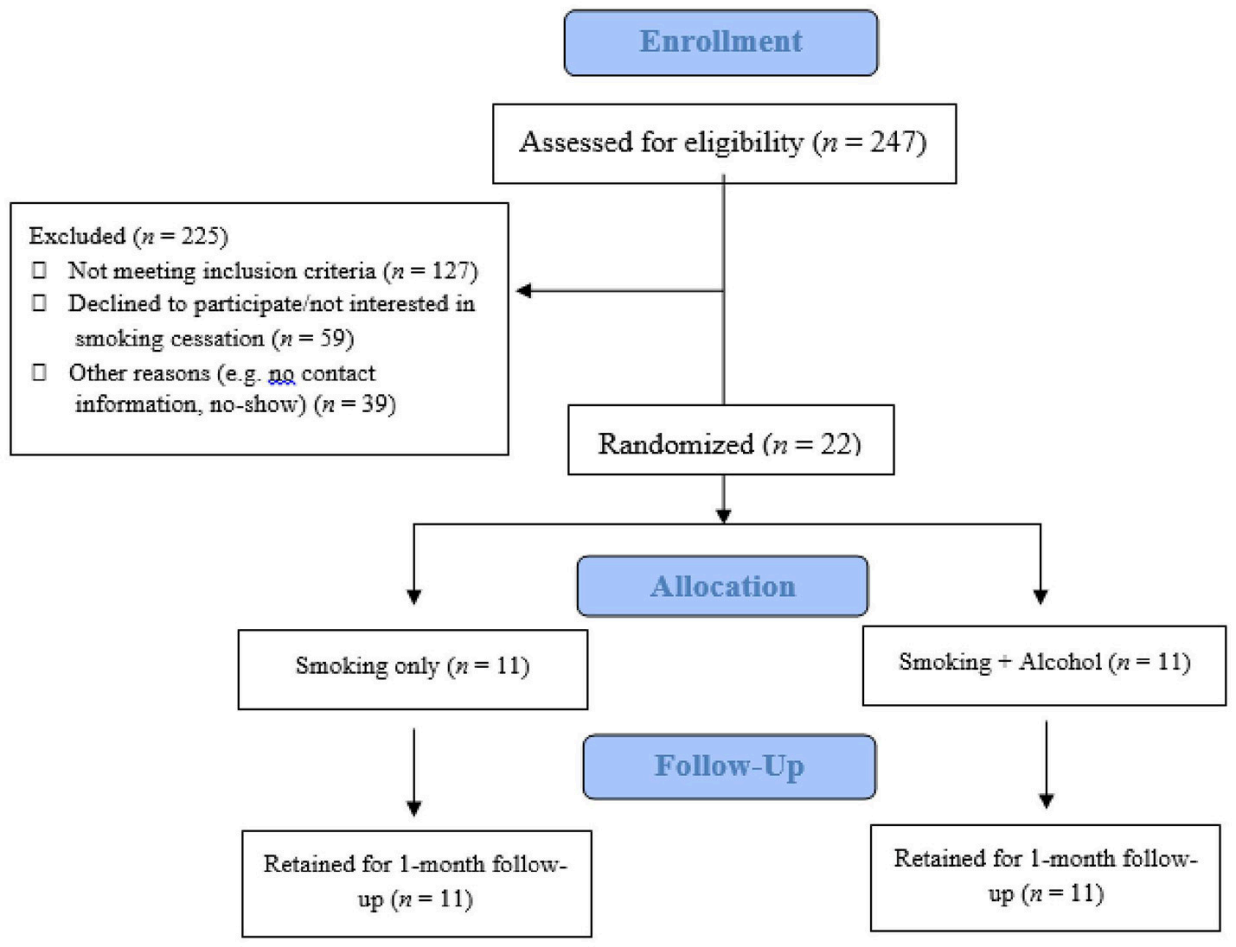
CONSORT diagram.

Eligible participants were randomly assigned to one of two counseling conditions: alcohol and smoking (SMK+A) condition or smoking only (SMK) condition (each condition *n* = 11; see Figure 1). Both conditions included motivational interviewing and brief behavioral counseling that lasted 50 min. Therapists were advanced clinical psychology graduate students supervised by a licensed clinical psychologist (LAR). During the counseling session, participants' motivation to quit smoking and reduce drinking was assessed using a Readiness to Change ladder [RTC; (33)]. Participants completed one 30-day follow-up session after the counseling session to assess alcohol and cigarette use, verified by CO and cotinine. At follow-up, participants completed questionnaires assessing how frequently they had used skills covered in the intervention (reported as number of times of use per skill within the past 30-day period), and how helpful each skill was in their cessation/reduction, based on a 5-point Likert scale (1 = “Not Helpful”; 3 = “Somewhat Helpful”; 5 = ”Very Helpful”), in addition to a follow-up RTC ladder. Participants were paid $20 at follow-up for attending both the intervention session and follow-up.

### Interventions

The 50-min smoking cessation counseling (SMK & SMK-A) interventions included four components that were based on effective motivational interview-based smoking and alcohol interventions (34–36). First, participants received personalized feedback on their cigarette use, including information on the number of cigarettes smoked daily, total number of smoking years, money spent on cigarettes, and quit attempts. Second, participants discussed pros and cons of smoking and quitting (“decisional balance”), as well as potential obstacles for quitting (e.g., cravings during specific times of the day). Third, participant and therapist worked together to set a smoking quit date on any day within the 30 days post-intervention. Lastly, a tailored skills-building approach was undertaken in which the therapist selected one skill (from a menu) for the participant, consistent with principles of motivational interviewing and brief behavioral counseling for substance use. The therapist's chosen skill was informed by the previous module discussing obstacles to quitting; the chosen skill aimed to help participants successfully cope with identified obstacles. Participants were also encouraged to select another two skills based upon his/her interests and goals. In the SMK condition, therapists selected a non-alcohol related skill, and the Reduce Alcohol Use skill was not an available skill for participants. However, in the smoking cessation counseling plus alcohol (SMK+A) condition, the therapist-selected skill was always the Reduce Alcohol Use skill. Therapists provided brief psychoeducation on the selected skills and encouraged participants to discuss and practice the skills in session. Therapists then worked with the participants to develop individualized plans that incorporated use of the skills outside of the session. The following non-alcohol related skills were available in the menu of options: (a) Get Support and Encouragement, (b) Identify and Cope with Urges, (c) Relaxation Techniques, (d) Gradual Cut Down, (e) Cigarette Refusal Skills, (f) Medication Guide, (g) Contact Quit Hotline, and (h) Access Online Resources.

For example, a participant in the SMK condition might have chosen the skills “Identify and Cope with Urges” and “Cigarette Refusal Skills,” in addition to the therapist's choice of “Medication Guide.” In this case, the therapist would begin the skills component of the intervention with information about abstinence- and cue-induced cravings, such as their temporary and undulating nature. The participant would have then been asked to identify recurrent situations in which they experienced craving. The therapist and participant would work to develop methods for coping with cravings, such as engaging in other activities, avoiding certain situations, or “urge surfing” (i.e., sitting with rather than fighting smoking cravings). The therapist and participant then would move on to discuss the second chosen skill, “Cigarette Refusal Skills,” which included a discussion on how to effectively communicate the participant's goals and needs with friends/family, and strategies for coping with resistance from others. Lastly, the skill of “Medication Guide” would be discussed, which would include general education on the purpose and function of the various approved medications for smoking cessation [e.g., nicotine replacement therapy (NRT), buproprion] and a discussion with the participant as to who to contact for more information and how to obtain medications (e.g., primary care physician, pharmacist).

The SMK+A condition was tailored to address alcohol use in several ways. First, the SMK+A condition included information on the participant's drinking patterns (e.g., drinking days per month and drinks per drinking day) during the personalized feedback component. Second, during the pros and cons discussion, participants in SMK+A additionally reviewed the pros and cons of reducing alcohol use while quitting smoking. Third, the therapist chose the “Reduce Alcohol Use” skill as one of the three skills to discuss during the skill selection component of the tailored intervention. This skill included education on the co-reinforcing properties of smoking and alcohol, discussion on identifying individualized high-risk situations that could lead to heavy drinking, and helped the participant plan feasible ways to reduce heavy drinking and smoking, such as staggering drinks at longer intervals, limiting cash to create a spending limit, and reducing availability of cigarettes during social drinking. Other than the addition of the Reduce Alcohol Use Skill, all skill items on the menu were identical to the SMK condition.

### Analytic plan

In accordance with NIH policies for and limitations of small feasibility trials in generating accurate and reliable effect sizes [NIH NOT-OD-16-149; (37, 38)], null hypothesis significance tests are not reported for clinical outcomes. *T*-tests and chi-square tests assessed potential demographic, skill use/acceptability, and substance use baseline differences between participants in each condition. Frequency of skill use and ratings of skill helpfulness are reported as indices of intervention acceptability, and participant retention and percentage of eligible participants among interested individuals were assessed for feasibility. Cessation based on 7-day point-prevalence at 30-day follow-up was operationalized as having a CO reading ≤ 4 parts per million (PPM) and cotinine levels ≤ 3 ng/mL, consistent with smoking cessation studies in the United States (39, 40). Also reported are pre-intervention and 1-month follow-up TLFB drinks per drinking day, number of heavy drinking days (greater than three drinks per day for women and four drinks per day for men), and RTC scores for smoking and alcohol use behaviors.

## Results

Demographic, behavioral, and substance use characteristics are presented in Table 1. Chi-square and *t*-tests revealed that there were no significant differences in any of these variables between the two intervention groups (*p*s = 0.36–0.68).

**Table 1:** Sample characteristics (*N* = 22).

**Variable**	**Full sample**	**SMK^a^**	**SMK + A^b^**
Age—M (SD)	38.5 (11.9)	40.1 (12.0)	36.9 (12.0)
**Gender—*****N*** **(%)**
Men	15 (71.4)	8 (72.7)	7 (63.6)
Women	7 (31.8)	3 (27.3)	4 (36.4)
**Race—*****N*** **(%)**
White	8 (36.4)	4 (36.4)	4 (36.4)
Black	10 (45.5)	4 (36.4)	6 (54.5)
Latino	6 (27.3)	3 (27.3)	3 (27.3)
**Substance use—M (*****SD*****), range**			
Fagerstrom Test for Nicotine	5.6 (1.6), 2–9	5.7 (1.6), 2–8	5.5 (1.7), 3–9
Dependence (FTND)
**Cigarette Smoking (30-day TLFB)**
Baseline cigarettes per day	14.2 (7.8), 5–40	12.6 (5.0), 5–20	15.9 (9.8), 7.6–40
Follow-Up cigarettes per day	2.1 (2.5), 0–9.4	2.2 (2.2), 0–6	1.9 (2.9), 0–9.4
Alcohol Use Disorders	14.3 (8.4), 4–34	13.9 (6.7), 6–24	14.7 (10.1), 4–34
Identification Test (AUDIT)
**Alcohol Use (30-day TLFB)**
Baseline drinks per drinking day	5.8 (4.9), 1.5–22	4.7 (2.3), 2.8–10.8	6.7 (6.3), 1.5–22
Follow-Up drinks per drinking day	3.5 (2.1), 1–8.7	3.5 (1.0), 2–5.3	3.5 (2.8), 0.3–8.7
Baseline heavy drinking days	11.3 (9.5), 0–30	11.1 (8.6), 0–27	11.5 (10.7), 0–30
Follow-Up heavy drinking days	3.2 (3.6), 0–13	3.8 (4.4), 0–13	2.6 (2.7), 0–9
**Readiness to change—alcohol**
Baseline	5.9 (3.0), 0–10	5.1 (3.1), 1–10	6.6 (2.8), 0–10
Follow-Up	6.4 (3.1) 0–10	5.3 (3.6), 0–10	7.7 (1.8), 5–10
**Readiness to change—smoking**			
Baseline	6.4 (1.7), 2–9	6.2 (1.9), 2–8	6.6 (1.5), 5–9
Follow-Up	7.3 (2.2), 2–10	6.8 (2.5), 2–10	7.8 (1.9), 5–10

a SMK = Smoking only intervention condition.

b *SMK+A = Smoking and alcohol intervention condition*.

### Feasibility

In total, ~25% of interested individuals were eligible for this study after initial phone screening. Of the 61 eligible individuals after the phone screening, 22 participants came to the laboratory for the in-person assessment and were randomized. This ratio of eligible to enrolled participants is consistent with recruitment rates in previous studies of treatment-seeking smokers (41). All 22 randomized participants were retained through 1 month follow-up (i.e., 100% retention rate). Together, the enrollment and retention rates suggest that this intervention is feasible in the context of an outpatient research clinic and in a community sample of treatment-seeking heavy drinking smokers. Transportability and feasibility in other health care settings, such as primary care clinics, warrant further investigation.

### Acceptability

Participants chose a quit date that ranged from 1 to 26 days post-intervention (M = 12.18, SD = 7.40), with no differences between intervention groups. Participants demonstrated variability in how frequently they used the skills discussed during the counseling session over the 30 days post-quit (0–300 instances; see Table 2). The most frequently utilized skill was breathing techniques and least frequently utilized skills were medications that reduce craving and quit hotlines. Within the SMK+A condition, participants reported considering the impact of their alcohol use approximately five times during the previous 30 days. On average, participants reported that most skills were between moderately and very helpful. Descriptively, participants rated “Get Support and Encouragement” and “Addressing Alcohol Use” as the most helpful skills, and rated “Medication Guide” and “Quitlines” as the least helpful skills.

**Table 2:** Utility of smoking cessation skills discussed in the brief counseling condition.

**Skill**	**How often did you use this skill in the last 30 days? M (*SD*), range**	**How helpful was this skill in the last 30 days? M (*SD*), range**
Breathing techniques	34.4 (70.2), 0–300	3.8 (1.3), 1–5
Identify and coping with urges	17.4 (14.6), 0–50	3.8 (1.3), 1–5
Get support and encouragement	14.3 (12.6), 0–30	4.0 (1.4), 1–5
Gradual reduction/cutting down	13.3 (13.0), 0–35	3.9 (1.2), 1–5
Dealing with social pressures	12.9 (14.2), 0–45	3.8 (1.1), 1–5
Addressing alcohol use^a^	4.7 (7.4), 0–25	4.1 (1.4), 1–5
Web resources	2.0 (5.5), 0–20	3.3 (1.7), 1–5
Medication guide	2.0 (3.8), 0–15	2.9 (1.7), 1–5
Quitlines	1.0 (2.3) 0–8	3.2 (1.8), 1–5

a *This item was only completed by participants randomly assigned to the smoking cessation + drinking reduction counseling condition (SMK+A)*.

At baseline, 6 of 11 SMK+A and 5 of 11 SMK participants reported a RTC smoking score ≥7, indicating intentions that range from being ready to quit smoking to starting to reduce cigarette consumption in preparation for quit. Such scores correspond with Preparation and Action stages of change (42). For alcohol use, 6 of 11 SMK+A and 4 of 11 SMK reported RTC alcohol scores greater than or equal to 7. One participant in each condition reported RTC alcohol scores of 10, indicating stable reductions in alcohol use that the participants wished to maintain.

At 1-month follow-up, 7 of 11 SMK+A and 4 of 11 SMK participants reported a RTC smoking score greater than or equal to 7, with 3 participants in each condition reporting scores of 10, or a desire to maintain smoking abstinence. Additionally, 2 participants in SMK+A reported RTC smoking scores of 9, indicating reductions in cigarette consumption in preparation for abstinence. For alcohol use, 6 of 11 SMK+A and 5 of 11 SMK participants reported a RTC alcohol score ≥7. Of these, 5 SMK+A and 3 SMK participants reported RTC scores of either 9 or 10; such participants had taken some action to reduce their drinking, and expressed a desire to cut back further and/or maintain these changes in drinking.

### Smoking and alcohol outcomes

**Figure 2:**
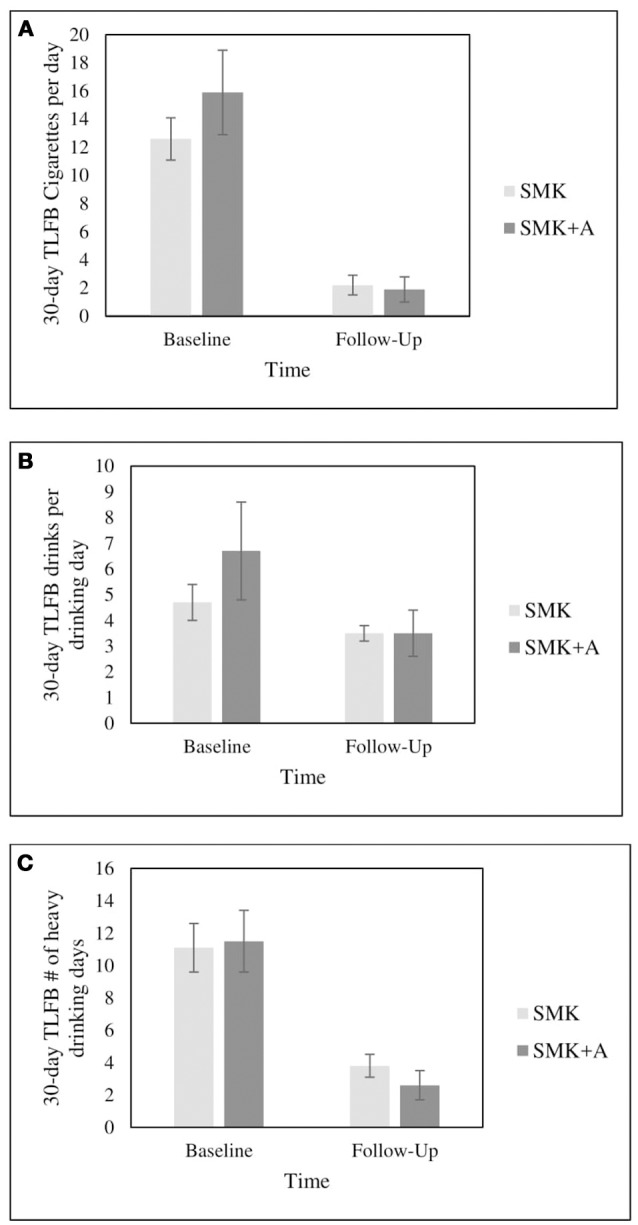
Comparisons over time of 30-day TLFB cigarettes per day **(A)**, drinks per drinking day **(B)**, and heavy drinking days **(C)**. Error bars indicate standard error.

A total of three participants in each intervention condition met criteria for bioverified 7-day point-prevalence smoking abstinence at 1-month follow-up. Visual comparisons by intervention group are available for 30-day TLFB cigarettes per day (see Figure 2A, drinks per drinking day (see Figure 2B), and heavy drinking days (see Figure 2C).

## Discussion

The current study examined acceptability and feasibility of a modified brief counseling intervention that incorporates alcohol reduction skills for heavy drinking smokers. Descriptive evidence supports both acceptability and feasibility of the modified intervention. One quarter of interested individuals were eligible after initial phone screening as treatment-seeking heavy drinking smokers, corroborating high comorbidity rates of cigarette smoking and drinking and potential applicability of the brief intervention for significant swaths of smoking populations. Combined with low dropout rate (0%) after randomization, these results demonstrate that recruitment and longitudinal retention of this subgroup of smokers for this brief intervention is feasible.

Acceptability was also demonstrated in this study through participant endorsement of skill utility. Addressing alcohol use in the SMK+A condition demonstrated the highest self-reported helpfulness score in achieving cessation and reduction goals. Although participants endorsed a range for how frequently they utilized each of the skills, the skills relating to utilization of quitlines and medications were reported to be the least helpful resources, and demonstrated the lowest frequency of utilizing these resources. Previous studies have documented the underutilization of these effective treatments among smokers (43, 44). Potential reasons for this underutilization include logistical barriers (i.e., monetary costs) (43), misconceptions about pharmacological treatments (45), and beliefs about quitting smoking unassisted (46). Therefore, if these results are replicated in larger studies, there may be potential benefit in discussing additional barriers regarding the utilization of these resources when clinically indicated.

Regarding RTC, a significant proportion of participants in both conditions reported sustaining or developing an interest in reducing cigarette smoking at 1 month follow-up. Participants in both conditions also expressed an elevated interest in reducing alcohol use, with over half of the participants in the SMK+A condition reporting that they have already taken steps to reduce alcohol use at 1 month follow-up. Previous studies have found that brief motivational interventions produce immediate changes in motivation for reducing cigarette use, but that these effects dissipate after 1 month (47). Though parametric pre-post comparisons were not made in this study due to small sample size, future studies that examine potential effects of the modified intervention on RTC for alcohol reduction are warranted; should such an effect be supported, it may be critical to take advantage of short-term increases in motivation for change. Supplementary assistance such as physician support (48) may be particularly beneficial in sustaining cessation/reduction attempts.

While the primary aim of the study was feasibility and treatment development, preliminary data were obtained and revealed that a meaningful number of participants successfully quit smoking following the brief intervention, with 6 out of 22 participants (27.3%) reporting successful biochemically verified quit at 1-month follow-up across both conditions. It is also promising that no participants in either condition reported an increase in cigarette smoking or alcohol use at follow-up. Previous work has suggested that level of alcohol consumption moderates the effects of smoking interventions with integrated alcohol components, such that moderate drinkers appear to benefit more from these interventions than heavy drinkers (12). Further, individuals with low levels of alcohol use disorder and lower alcohol consumption reduce their drinking even after brief counseling (49), suggesting the present intervention may be particularly relevant for heavy-drinking smokers with lower alcohol consumption and use disorder severity.

The most unique strengths of this intervention include its brief duration, practicality, feasibility, low cost, and the ease with which it can be incorporated within various types of settings (e.g., primary care, residential substance use treatment, urgent care) and administered by myriad health service professionals (e.g., nurses, counselors, tobacco treatment specialists, chemical dependency counselors). Limitations of the current study include its small sample size that preclude reliable effect size estimates and reinforce the need for replication, the absence of a control condition to control for quit rates attributable to study enrollment, and lack of quantitative assessment regarding participant's chosen smoking cessation skills. Future studies that examine and further develop these treatments in multiple settings, particularly in the context of combined treatments, may be important to understand whether this intervention provides compounded benefits in sustaining motivation for smoking and alcohol reduction. For instance, though there are mixed results regarding the efficacy of naltrexone in increasing smoking abstinence and decreasing alcohol use (50, 51), there is emerging evidence that a combination of naltrexone and varenicline may reduce use of both cigarettes and alcohol among heavy drinking smokers (52). Incorporation of brief counseling sessions into studies of these effective medications may therefore be a promising treatment venue to reinforce such healthy behavior change for these individuals in different treatment settings.

## Conclusions

This initial study provides preliminary feasibility and acceptability data on a brief counseling intervention for smoking cessation targeted for heavy-drinking smokers. Discussing the use of alcohol reduction in the context of motivational-interview based smoking cessation counseling was overall deemed acceptable and feasible. Larger studies will help to elucidate this intervention's effects on readiness to change, smoking cessation, and alcohol reduction. Such work that builds upon current practice guidelines for heavy-drinking smokers may be crucial for this difficult to treat population.

## Ethics statement

This study was carried out in accordance with the recommendations of University of California Los Angeles Office of the Human Research Protection Program with written informed consent from all subjects. All subjects gave written informed consent in accordance with the Declaration of Helsinki. The protocol was approved by the University of California Los Angeles Institutional Review Board.

## Author contributions

CL conducted data analyses and wrote the first draft of the manuscript. NM, KC, and VA acquired study data, developed hypotheses, and conducted preliminary analyses. AML and LR provided critical revisions on all manuscript sections. LR also conceived of the study and its design. All authors contributed extensively to the manuscript.

### Conflict of interest statement

LR has served as a paid consultant for GlaxoSmithKline and received study medication from Pfizer. The remaining authors declare that the research was conducted in the absence of any commercial or financial relationships that could be construed as a potential conflict of interest.
